# Supplementary material for: Identification of photoperiod-induced specific miRNAs in the adrenal glands of Sunite sheep (Ovis aries)
Source: Front Vet Sci. 2022 Jul 22;9:888207. doi: 10.3389/fvets.2022.888207 (PMC9354845; doi:10.3389/fvets.2022.888207)
Supplement: Supplementary file 1 [file Data_Sheet_1.ZIP › Supplementary file/miRDeep2 file.zip!/miRDeep2 file/Novel_Structure/LP42A1/Novel_327.pdf]

Provisional ID	:	NC_019484.2_35644
Score total	:	16.4
Score for star read(s)	:	3.9
Score for read counts	:	8.8
Score for mfe	:	2.2
Score for randfold	:	1.6
Score for cons. seed	:	
Total read count	:	29
Mature read count	:	28
Loop read count	:	0
Star read count	:	1

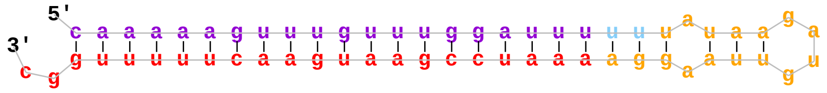

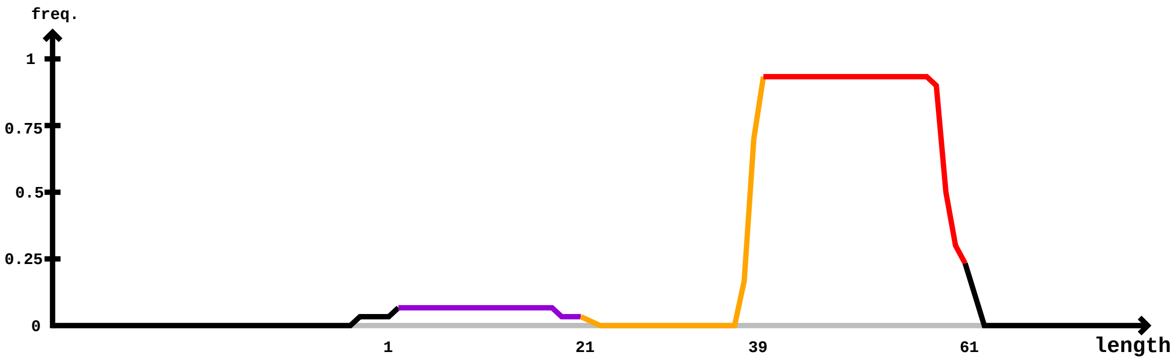

Star Mature

```
5'- uacaugugcgaauuguuacuaauauugggcuggccaaaaaguuuguuuggauuuuuuauaagauguuaaggaaaauccgaaugaacuuuuuggccugcccaauacaucugcc   -3'    obs
    uacaugugcgaauuguuacuaauauugggcuggccaaaaaguuuguuuggauuuuuuauaagauguuaaggaaaauccgaaugaacuuuuuggccugcccaauacaucugcc          exp
    ......................(((((((.(((((((((((((((((((((((((((.(((....))).)))))))))))))))))))))))))).))))))))........   reads      mm         sample
    ..............................uggccaaaaaguuCguuugga.............................................................   1          1          seq
    ..................................caaaaaguuuguuuggGuuu..........................................................   1          1          seq
    ......................................................................gaaaauccgaaugaacuuuu......................   1          0          seq
    ......................................................................gaaaauccgaaugaacuuuuu.....................   1          0          seq
    ......................................................................Aaaaauccgaaugaacuuuuu.....................   1          1          seq
    ......................................................................gaaaauccgaaugaacuuuuug....................   2          0          seq
    .......................................................................aaaauccgaaugaacuuuuu.....................   7          0          seq
    .......................................................................aaaaucAgaaugaacuuuuu.....................   1          1          seq
    .......................................................................aaaauccAaaugaacuuuuu.....................   2          1          seq
    .......................................................................aaaauccgaaugaacuuuuug....................   3          0          seq
    .......................................................................aaaauccgaaugaacuuuuuU....................   1          1          seq
    .......................................................................aaaauccgaaugaacuuuuugg...................   2          0          seq
    ........................................................................aaaCccgaaugaacuuuuuggc..................   7          1          seq
```
